# Reducing the false positive rate in the non-parametric analysis of molecular coevolution

**DOI:** 10.1186/1471-2148-8-106

**Published:** 2008-04-10

**Authors:** Francisco M Codoñer, Shirley O'Dea, Mario A Fares

**Affiliations:** 1 Evolutionary Genetics and Bioinformatics Laboratory, Department of Genetics, Smurfit Institute of Genetics, University of Dublin, Trinity College, Dublin, Ireland; 2 Institute of Immunology, Department of Biology, National University of Ireland, Maynooth, Ireland

## Abstract

**Background:**

The strength of selective constraints operating on amino acid sites of proteins has a multifactorial nature. In fact, amino acid sites within proteins coevolve due to their functional and/or structural relationships. Different methods have been developed that attempt to account for the evolutionary dependencies between amino acid sites. Researchers have invested a significant effort to increase the sensitivity of such methods. However, the difficulty in disentangling functional co-dependencies from historical covariation has fuelled the scepticism over their power to detect biologically meaningful results. In addition, the biological parameters connecting linear sequence evolution to structure evolution remain elusive. For these reasons, most of the evolutionary studies aimed at identifying functional dependencies among protein domains have focused on the structural properties of proteins rather than on the information extracted from linear multiple sequence alignments (MSA). Non-parametric methods to detect coevolution have been reported to be especially susceptible to produce false positive results based on the properties of MSAs. However, no formal statistical analysis has been performed to definitively test the differential effects of these properties on the sensitivity of such methods.

**Results:**

Here we test the effect that variations on the MSA properties have over the sensitivity of non-parametric methods to detect coevolution. We test the effect that the size of the MSA (number of sequences), mean pairwise amino acid distance per site and the strength of the coevolution signal have on the ability of non-parametric methods to detect coevolution. Our results indicate that all three factors have significant effects on the accuracy of non-parametric methods. Further, introducing statistical filters improves the sensitivity and increases the statistical power of the methods to detect functional coevolution. Statistical analysis of the physico-chemical properties of amino acid sites in the context of the protein structure reveals striking dependencies among amino acid sites. Results indicate a covariation trend in the hydrophobicities and molecular weight characteristics of amino acid sites when analysing a non-redundant set of 8000 protein structures. Using this biological information as filter in coevolutionary analyses minimises the false positive rate of these methods. Application of these filters to three different proteins with known functional domains supports the importance of using biological filters to detect coevolution.

**Conclusion:**

Coevolutionary analyses using non-parametric methods have proved difficult and highly prone to provide spurious results depending on the properties of MSAs and on the strength of coevolution between amino acid sites. The application of statistical filters to the number of pairs detected as coevolving reduces significantly the number of artifactual results. Analysis of the physico-chemical properties of amino acid sites in the protein structure context reveals their structure-dependent covariation. The application of this known biological information to the analysis of covariation greatly enhances the functional coevolutionary signal and removes historical covariation. Simultaneous use of statistical and biological data is instrumental in the detection of functional amino acid sites dependencies and compensatory changes at the protein level.

## Background

There has been great interest in understanding the role of amino acid covariation in protein evolution and function. The importance of covariation goes beyond the linear contribution of amino acid coevolution to explain the stability of protein folds. In fact not only conservation of amino acid positions may be important for maintaining protein folds but also the correlated variation of pairs of amino acid residues around these folds may have an important role in maintaining their stability [1]. Covariation may therefore reflect functional and/or structural constraints. Many parametric and non-parametric methods have been developed for such purpose (i.e., [2-12]). Methods based on the information theory are among the most used ones. These methods measure the reduction in uncertainty in particular amino acid sites (entropy) achieved by considering the site's mutual information (MI) with additional sites. This measure accounts for the predictability of the composition of one amino acid column when the composition of another amino acid site is known. However, the sensitivity of most of these methods has been always halted by the fact that true positive coevolutionary relationships between amino acid sites are swamped in a background of stochastic amino acid covariation. Consequently, studies using methods slightly different in their mathematical parameterisation have been dramatically affected by the properties of MSAs (i.e., Number of sequences in the alignment, mean pairwise sequence amino acid distances, etc.). In addition to the problems of stochastic covariation between amino acid sites, no single pair of sites is unrelated in MSAs due to their historical (phylogenetic) dependencies [6,7,13]. Finally, the coevolutionary relationships are due to many different links between amino acid sites including coevolution due to structural, functional or physical interactions [14].

Many authors have devised mathematically transformed models based on the mutual information of two amino acid sites to highlight true functional coevolutionary relationships between them. Some of these methods included a correction of MI values by the degree of amino acid variability at the amino acid sites [15], using the phylogenetic information to remove phylogenetic coevolution [6,7,16], limiting the size of groups of coevolving amino acid sites [13] or quantitatively normalising the MI value by the content of information at the sites under test [17]. Even though most of these methods have improved the sensitivity to detect functional/structural relationships between coevolving amino acids, a realistic method accounting for some of the most obvious biological properties of amino acid sites is as yet to be performed. For instance, most of the methods developed use qualitative approaches to remove the phylogenetic coevolutionary signal [i.e., [12,7]]. A formal or statistical method is then needed to conduct a more rigorous approach of removing the undesired historical coevolution.

Introducing some biological parameters, such as the physico-chemical properties of amino acid sites may dramatically increase the sensitivity of mutual-information based methods to detect coevolution. Furthermore, these biological-information based methods may improve our ability in detecting compensatory mutations. This is due to the fact that compensatory mutations usually occur proximal in the protein structure and they are produced to maintain internal volumes, preserve salt bridges, or retain optimal hydrogen bonding. In summary, information-based coevolutionary analyses introducing biological information may indirectly aid at identifying pairs of three-dimensionally proximal residues. Few attempts have been made to relate coevolving pairs in a MSA to biological information in order to shed light on the structural and functional reasons of such coevolution [2,6,7,18,19]. These reports however, did not use this biological information as a filter for the coevolutionary analyses but rather as a criterion adopted *a posteriori *to performing the method to detect functionally important coevolving pairs. Also, no general approach was conducted to determine the relationships between such parameters and their biological meaning. Because of the growing interest in detecting molecular coevolution to infer biologically meaningful selective constraints, measuring the accuracy of such methods when varying the MSAs properties becomes crucial.

In this work we first examine the evolutionary and statistical behaviours of some of the properties of amino acid sites in proteins. Then we introduce this information in the coevolutionary analysis and test its effect on the sensitivity of MI-based methods. Because of the large amount of data examined in this study, we implemented these MI methods in in-house software (available from the corresponding author on request). We then examined the effect of MSAs with different properties. Finally, we apply our improved procedure to detect coevolution to several well-studied proteins, whose functional domains are known and examine the ability of the method to detect important functional sites.

## Results

### Filtering by the parsimony information criterion removes most of the stochastic coevolution

**Figure 1:**
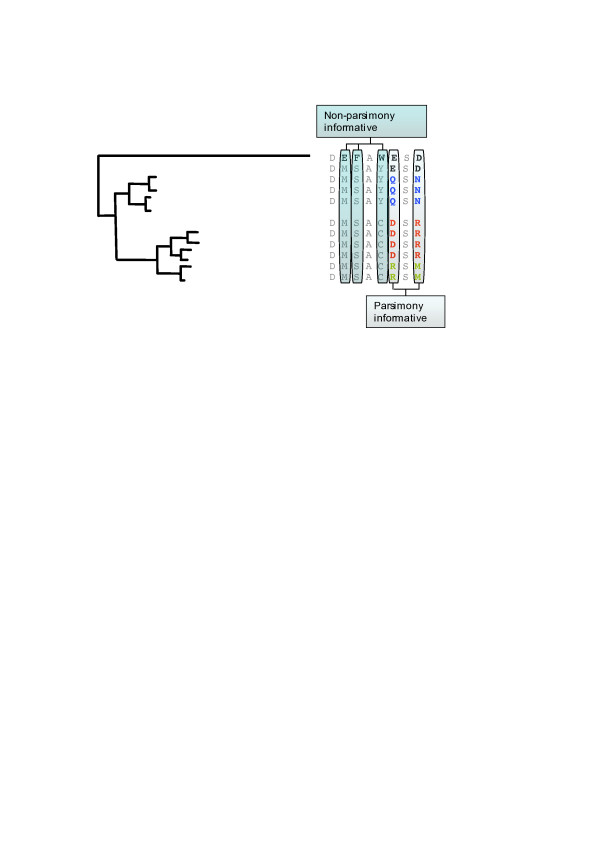
**Identification of pairs of parsimony-informative coevolving amino acid sites**. The figure represents a multiple sequences alignments with the phylogenetic relationships of its constituent sequences. The phylogenetic tree is asymmetric due to the accelerated rates of evolution of one of the sequences (long branch). As a result, most of the evolutionary signal (amino acid variability) in the multiple protein sequence alignment (right hand) is due to amino acid differences at sites contributed by the accelerated sequence. Parsimony-informative coevolving sites (those sites showing at least two different amino acid states with each one represented by at least one sequence of the alignment) are shown, and the variability in their states is colour coded. Non-parsimony informative coevolving sites are also highlighted as example of phylogenetic coevolution.

**Figure 2:**
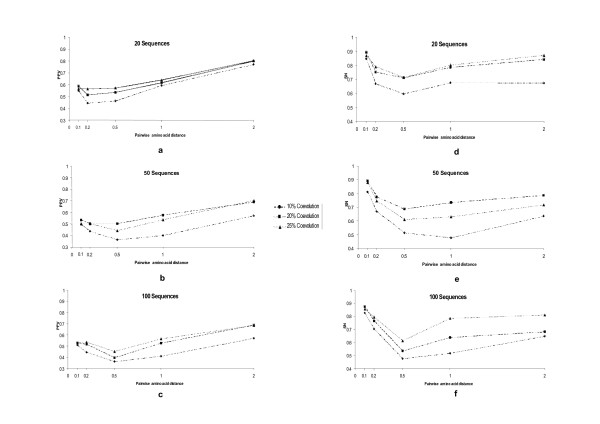
**Testing the effect of different parameters in the percentage of positive values (PPV, plots a to c) and sensitivity (SN, plots d to f) of Mutual Information Criterion based methods to detect coevolution when parsimony filtering is applied**. We tested the effect on PPV and SN of variations in the size of the multiple sequences alignment (MSAs, with the sizes ranging between 20 and 100 sequences), mean pairwise amino acid distance (with distances ranging between 0.1 and 2 amino acid substitutions per site) and strength of coevolution (the level of coevolution ranged between a minimum of 10% and a maximum of 25%). Coevolution levels indicate the distribution of patterns of coevolution at a particular pairs of amino acid sites. For example levels of 10% indicate that 10% of sequences at that particular pair of sites in the multiple sequence alignment correlate in one amino acid state pattern whereas the 90% remaining correlate in a different state. The strongest levels of coevolution (those showing highest MI values) will be presented by those pairs of sites showing 25% of coevolution strength.

One of the pre-requisites to consider a site as valid for our analyses was when it presented enough information as to remove the effects of the phylogenetic asymmetry on the data. For example, if one particular sequence were significantly distant from the root of the tree when compared to other sequences, then its effect on the amount of evolutionary information over the data would be more significant before any correction was applied. To account for the asymmetric contribution of sequences to the coevolutionary analysis we used a parsimony criterion to filter down the number of pairs of sites considered in the analysis. A site was considered parsimony informative if the number of amino acid states at that site was greater than two and if each state is present in at least two sequences (see Material and Methods for details and Figure 1). This approach has previously been shown to reduce the false discovery rate [18]. We first tested the effect of filtering by parsimony in MI values at different coevolutionary strengths, differently populated MSAs and at various amino acid distances. One of the most important results obtained in this analysis was that the percentage of positive values (PPV; see Material and Methods for description) increases from a maximum value of approximately 20% in a previous work where no filter was applied [7] to a maximum value of 82% when using 20 sequences, and 80% when using 50 and 100 sequences (Figure 2). This important increase in PPV value suggests that amino acid distances per site are an important factor to take into account when performing this kind of approaches as previously suggested [17]. The sensitivity values for this non-parametric method are also of the same order than that yielded by other parametric methods shown to be highly sensitive [7]. A univariate analysis of variance shows significant effects of the number of sequences (*F*_2 _= 37.912; P < 0.001); mean pairwise amino acid substitutions per site (*F*_4 _= 150.266; P < 0.001); and strength of coevolution (*F*_2 _= 118.282; P < 0.001) on the PPV values. The pairwise factors' interactions and the interaction between the three factors also showed significant effects on the PPV values. Inspection of the mean PPV values in MSAs with different sizes did not show a clear tendency when varying the number of sequences (Figure 2a to 2c). This result may be due to the fact that introducing the parsimony filter makes the MI based method more robust to variations in the MSAs sizes due to a lower effect of the stochastic and phylogenetic covariations. Increasing the pairwise divergence levels or the strength of coevolution increased the mean PPV values (especially when comparing PPV values in MSAs with 10% coevolution to those with 20% and 25% coevolution) (Figure 2a to 2c).

Comparison of coevolution data corresponding to the levels of 20% and 25% coevolution presented however no significant differences for both sets (*F*_1 _= 0.732; *P *= 0.392). The interaction between the level of coevolution (20% and 25%) and the variation of pairwise divergence levels also showed no effect on the detection of true coevolution (*F*_2 _= 1.061; *P *= 0.374). Neither the interaction between these three factors showed any significant variability levels in PPV (*F*_8 _= 1.279; *P *= 0.250). However, pairwise distance as well as number of sequences still showed significant effects on the variance of PPV, with increasing pairwise distance being the only factor explaining a significant increase in PPV. Regarding the sensitivity (SN) of the method when filtering by parsimonious variability per site, all three factors (number of sequences, strength of coevolution and pairwise divergence levels) as well as all possible combination of factors influenced the variance of SN. In fact, univariate analyses showed significant effect of the size of the MSA (*F*_2 _= 105.99; *P *< 0.001), amino acid substitutions per site (*F*_4 _= 223.775; *P *< 0.001) and strength of coevolution (*F*_2 _= 89.214; *P *< 0.001) on SN. The tendency of SN with variations of each of the factors was however different (Figure 2d to 2f). Even though an increase of the number of sequences from 20 to 50 influenced the SN and PPV values, further increasing to 100 sequences in the MSA did not show any directional influence in SN as it remained within the same range of values (Figure 2d to 2f). When distances were greater than 0.1 amino acid substitutions per site SN tended to increase with increasing coevolutionary signal and increasing pairwise amino acid distances (Figure 2d to 2f). As before, the effect was more pronounced when the signal of coevolution increased from 10% to 20% or to 25% coevolution. In fact, significant differences were observed between the dataset belonging to the 10% of coevolution level and that belonging to the 20% coevolution level (*F*_1 _= 130.339; *P *< 0.001). No differences were found however between the 20% and 25% datasets (*F*_1 _= 1.126; *P *= 0.289). Our results indicate that strong signals of coevolution ameliorate the effect of the number of sequences and pairwise amino acid distances. This was especially notorious in the case of pairwise distance factor because its interaction with coevolution level factor shows no significant variability in SN (*F4 *= 0.963; *P *= 0.427). Finally, at high number of sequences, a direct relationship exists between the level of coevolution and the PPV values. For instance the mean PPV value increased when we increased the strength of coevolution from 10% to 20% in MSAs comprising 100 sequences (*F1 *= 42.325; *P *< 0.001). This increase is also significant when coevolution increases from 20% to 25% (*F1 *= 4.124; *P *= 0.043). We obtained the same results when we tested the effect of the strength of coevolution on SN. Also, there was no proportionality at low number of sequences between the level of coevolution and the PPV and SN values (an increase in the coevolution level does not imply a proportional increase in PPV or SN values).

### Biological filtering increases the robustness of the sensitivity of coevolution analyses

**Figure 3:**
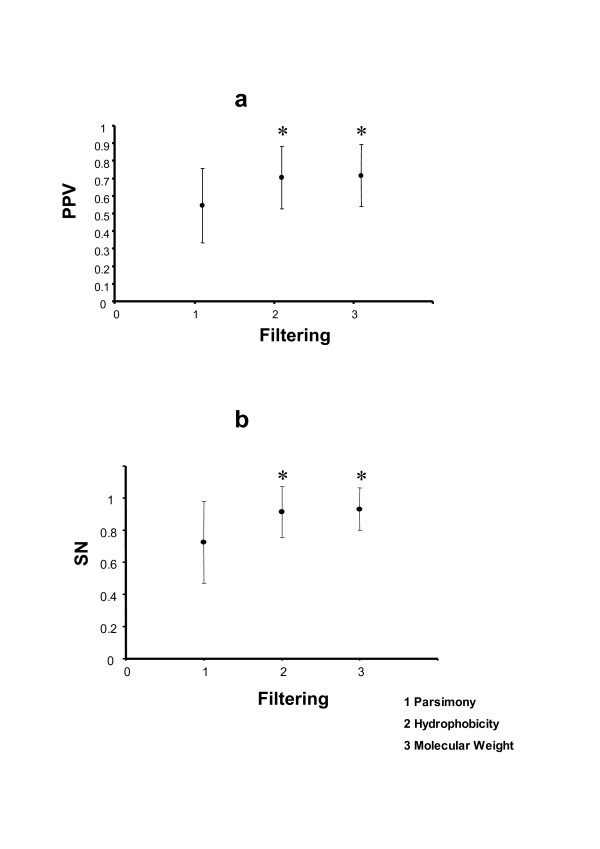
**Test of the improvement of the percentage of positive values (PPV) detected and sensitivity (SN) when we add filters to the detection of coevolution**. The X-axis represents the different filters (1 = parsimony, 2 = hydrophobicity and 3 = molecular weight). Y-axis represents PPV (a) or SN (b). The comparison of each filter against filtering by parsimony was always significant (* indicates P < 0.01).

One of the main purposes of coevolutionary analyses is to highlight covariation produced by biologically meaningful relationships between sites. Two of such important biological properties influencing the distribution of sites in the protein structure are the hydrophobicity and the molecular weight of the corresponding amino acids. We tested the relationships between amino acid sites in a set of 8,000 proteins whose structures were available in the National Center for Biotechnology Information (NCBI) based on these two factors. We then tested whether the correlation in these factors depends on the proximity of the pair of amino acid sites in the protein structure. To test this hypothesis, we measured the Euclidean atomic distance between all pairs of amino acids within a protein and categorised these distances into the following classes (6 Å, 9 Å, 12 Å, 15 Å, 18 Å, 21 Å, and more than 21 Å). This classification includes amino acids that are in physical contact in the structure (those which maximum distance is of 6 Å), those that interact through an intermediate amino acid (amino acids which distance ranges between 6 and 9 Å) and so on and so forth. We finally, obtained the average correlation values for the hydrophobicity and Molecular weight variability for each category (see Methods for details) and we analysed the variation in the correlation coefficients. For each protein structure we used a minimum of 10 protein sequences isolated from phylogenetically related eukaryotic species. The analysis of the 8000 protein structures showed a strong negative correlation between atomic amino acid distance and covariation at these parameters (For molecular weight: *ρ*_spearman _= -0.964, *P *<< 0.001; for hydrophobicity: *ρ*_spearman _= -0.750, *P *< 0.05). These results clearly show that correlation between amino acid sites using these amino acid properties would be useful to identify proximity between sites and hence may aid in identifying the source of amino acid covariation (for example, distinguish between long-range distance covariation from structural covariation or compensatory covariation). When testing the effect of hydrophobicity and molecular weight compared to parsimony filtering criterion in simulated MSA data, we found that introducing biological information increases the mean PPV and SN compared to parsimony filtering (Figure 3). The mean PPV increases significantly when we compare hydrophobicity to parsimony (*t*_8981 _= 38.474; *P *< 0.001) and when we compare molecular weight to parsimony (Mw) (*t*_8981 _= 41.491; *P *< 0.001) (Figure 3a). SN also increases significantly in both comparisons (*t*_8981 _= 42.076; *P *< 0.001 and *t*_8981 _= 47.952; *P *< 0.001, for the comparison of parsimony to hydrophobicity and to molecular weight, respectively) (Figure 3b).

The PPV values generated in the coevolution analyses filtered by the correlated hydrophobicity of the pairs of amino acid sites were influenced by all three factors: size of MSAs, mean pairwise distance and level of coevolution. However, simultaneous variation of the coevolution levels and size of MSAs or mean amino acid pairwise distance showed no effect on the amount of true positive values detected, with high coevolution levels compensating for variations in the MSA size (*F*_4 _= 0.186; *P *= 0.946) or mean pairwise amino acid distance (*F*_8 _= 0.880; *P *= 0.533). The combination of the three factors did not have significant effect on the PPV values generated (*F*_16 _= 0.962; *P *= 0.496).

**Figure 4:**
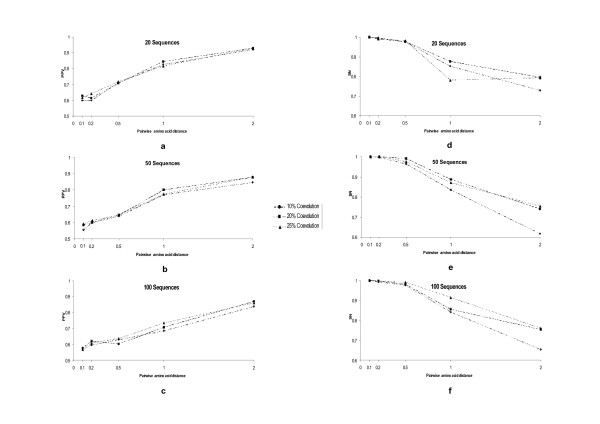
**Testing the effect of different parameters in the percentage of positive values (PPV, plots a to c) and sensitivity (SN, plots d to f) of Mutual Information Criterion based methods to detect coevolution when filtering by the correlation in hydrophobicity is applied**. We tested the effect on PPV and SN of variations in the size of the multiple sequences alignment (MSAs, with the sizes ranging between 20 and 100 sequences), mean pairwise amino acid distance (with distances ranging between 0.1 and 2 amino acid substitutions per site) and strength of coevolution (the level of coevolution ranged between a minimum of 10% and a maximum of 25%).

**Figure 5:**
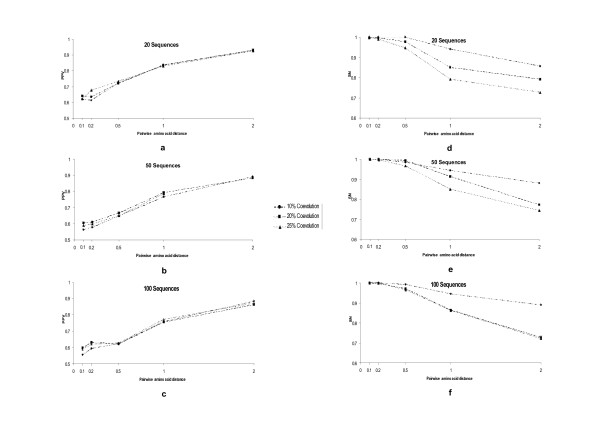
**Testing the effect of different parameters in the percentage of positive values (PPV, plots a to c) and sensitivity (SN, plots d to f) of Mutual Information Criterion based methods to detect coevolution when filtering by the correlation in molecular weight is applied**. We tested the effect on PPV and SN of variations in the size of the multiple sequences alignment (MSAs, with the sizes ranging between 20 and 100 sequences), mean pairwise amino acid distance (with distances ranging between 0.1 and 2 amino acid substitutions per site) and strength of coevolution (the level of coevolution ranged between a minimum of 10% and a maximum of 25%).

In examining the tendency of PPV at different coevolutionary levels, MSAs sizes and mean pairwise amino acid distance, PPV values increased with increasing amino acid distances (*F*_4 _= 13.726; P < 0.001) (Figure 4a to 4c). SN values in contrast decreased with increasing pairwise amino acid distances (Figure 4d to 4f). Variation in the size of the MSA had no effect on the SN values (*F*_2 _= 1.721; *P *= 0.179). Molecular weight also increased significantly the PPV and SN of the method to detect coevolution (Figure 5). Even though all three factors have significant effects on the PPV values, at high pairwise amino acid distances, PPV values are indistinguishable when using different coevolution levels (*F*_2 _= 0.092; *P *= 0.912) (Figure 5a to 5c). On the other hand, the PPV values increases with increasing MSAs sizes (*F*_2 _= 92.807; *P *< 0.001) and increasing mean pairwise distances (*F*_4 _= 815.347; *P *< 0.001). In the case of SN values, these were highly affected by all three parameters, although the number of sequences had little effect on the SN outcome (Figure 5d to 5f).

### Filtered coevolution analyses identify functional sites in the proteins GroEL, Hsp90 and Env

**Figure 6:**
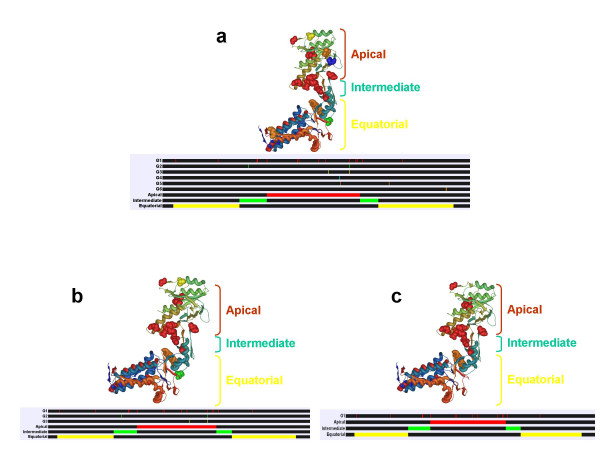
**Example of the effect of biological filters in coevolution analyses. In this case study we have used the heat-shock protein GroEL, which functional domains are well characterised**. The different domains (apical, intermediate and equatorial) are identified in the cartoon representing the linear GroEL sequence in red, green and yellow colours, respectively. G1 to G6 blocks show the sites under coevolution belonging to each coevolution group as coloured bars. Sites belonging to the same group of coevolution are shown in the crystal structure of one of the GroEL subunits (PDB accession number: 1svt.pdb) and are coloured following the same pattern as in the blocks of the groups of coevolution.

To test the performance of the method in detecting functionally important sites (see for example functional sites reported for GroEL in Table 1) or sites surrounding functional domains we used a MSA for the heat-shock proteins GroEL and Hsp90 and the envelope protein env from HIV-1. We were interested in pairs of amino acid sites that are either functionally important themselves or that are alternatively surrounding important functional domains (less than 8 Å distant from functionally important sites that have been described in other works, see Material and Methods for details). In the protein GroEL we identified 866 pairs of amino acid sites with significant MI values. Parsimony filtering reduced the number of pairs to 110 including sites organised into 6 groups of coevolution (Table 2). Group G1 was the largest coevolution group and comprised 14 amino acid sites, each one of them coevolving with the rest of the sites in the group. Out of the 14 amino acid sites detected in the first group, 3 amino acids (D11, V94, C458) were located in the equatorial domains, 3 amino acids (K171, E172, I379) in the intermediate domain and 8 amino acids (E191, A251, V254, A293, G306, E354, Q366, V369) in the apical domain (Figure 6a). Here, all amino acid numbers refer to the amino acid position in the reference GroEL sequence from the bacterium *Escherichia coli *K12. Although linearly scattered, these sites were concentrated in the three-dimensional structure of GroEL around three main functional domains (Figure 6a). Two amino acid sites were surrounding (less than 8 Å distant) the ATP binding region, one amino acid site (D11) could not be assigned to any functional category or was not proximal to any functional domain in the three-dimensional structure and the rest of amino acid sites (11 out of the 14) were enveloping the substrate/GroES binding domain (Figure 6a). In addition, we measured the atomic distances between any pair of proximal amino acids within group 1. This measurement was done assuming that effects of amino acid changes would be transmitted from one amino acid to another with which would coevolve following the shortest amino acid pathway. The shortest distances between any pair of coevolving amino acids within group 1 had as average 7.45 Å and ranged between 1.33 Å and 10.9 Å, indicating very short distances between coevolving amino acids. Five other groups (G2 to G6) each with a single pair of coevolving amino acid sites were also detected, with only G2 and G3 including a single amino acid (E354) present in both groups and proximal to functional regions (Table 2). All these groups, except group G4, presented very large atomic distances between the coevolving amino acids (all greater than 40 Å).

**Table 1:** Amino acid sites in *Escherichia coli *detected to be important for the function of GroEL.

Amino acid site	Function Description	Literature
30–33, 51–53, 87–91, 150–151, 398, 414–416, 454, 478–481, 493, 495	ATP/ADP and Mg^2+ ^binding residues (ATP/Mg-B)	[48]
199, 201, 203–204, 234, 237, 259, 263–264	Substrate binding (SubB)	[48]
230, 238, 241, 257, 260, 261, 265, 268, 270, 271	Substrate binding(SubB)	[49]
234, 237, 238, 241, 242, 257, 261, 265, 270	GroES binding (ESB)	[48]
241, 257, 261, 265, 270	GroES binding (ESB)	[49]
4, 41–42, 58–59, 61, 63, 75–76, 80, 83, 178–179, 188, 196–197, 224–226, 252, 253, 255, 257, 277, 283, 286, 303, 304, 308, 327, 328, 359, 361, 363, 364, 367, 368, 371, 380, 386, 390, 393, 397, 404, 408, 523	Charged residues exposed to the central cavity in the cis ring, probably contacting substrates (CER)	[50]

**Table 2:** Intra-molecular coevolution analysis in the heat-shock protein GroEL.

Group of coevolution	**Amino acid sites**^a^	**Mean H(X)**^b^	**Mean H(Y)**^c^	**Mean H(X, Y)**^d^	**Mean MI**^e^
G1	D11, V94, C458, K171, E172, I379, E191, A251, V254, A293, G306, E354, Q366, V369	0.223	0.238	0.244	0.217
G2	S154, E354	0.231	0.319	0.319	0.231
G3	A312, E354	0.324	0.319	0.439	0.204
G4	D334, A530	0.202	0.202	0.202	0.202
G5	V336, Q432	0.473	0.394	0.624	0.243
G6	M544, G545	0.231	0.231	0.231	0.231

^a ^Amino acid sites included in each coevolution group. All sites within the same group coevolve between each other. Numbers indicate the position of the site taking as reference the sequence of the bacterium *Escherichia coli *K12.

^b ^Measure of mean uncertainty for the first site of the coevolving pair.

^c ^Measure of mean uncertainty for the second site of the coevolving pair.

^d ^Measure of the mean joined uncertainty for the pairs of sites within each group

^e ^Mutual Information mean value for all the possible coevolving pairs within the group.

Application of the filter of hydrophobicity reduced the number of groups from 6 to 3 groups, including G1 to G3 (Figure 6b). Interestingly, All these groups included sites proximal to functionally important regions, although G2 and G3 only included one out of the two amino acid sites to be proximal (less than 8 Å distance) to functional regions.

Finally, Only G1 was detected when we applied the filter of correlation in the variance of the molecular weight or molecular weight and hydrophobicity between the coevolving amino acid sites (Figure 6c). Once again, only the group sharing the greatest number of functionally/structurally important sites was identified. In conclusion, the results show that application of variation in the biological properties of coevolving amino acids *a priori *identified removes most of the non-biologically relevant pairs of significant coevolving amino acids.

In the case of Hsp90 and env proteins' analyses we have not used an illustrative figure for the sake of synthesis and because the structure for env protein was lacking. Hsp90 comprises 4 main functional domains, including the ATP-binding domain (going from amino acid site 1 to 236), the flexible charged linker (between amino acid sites 237 and 271), the catalytic middle domain (between amino acids 272 and 617) and the C-terminal domain (between amino acids 618 and 732) involved in dimerisation [20]. We identified 1645 pairs (0.61% of all the possible pairs of sites) of amino acid sites with significant MI values at a significant level of 0.001 in Hsp90. Application of the parsimony filter reduced the number of pairs to 13 pairs of amino acid sites organised into 13 groups (G1 to G13) of coevolution (Table 3). Many of these groups (G2, G3, G6 and G7) included amino acid sites (E244 in G2 and G3; V260 in G6) located in the charged linker domain involved in client protein binding or surrounding sites important for client protein binding (A287 in G7, that is around P320) [20]. Other amino acid sites were located in or three-dimensionally close to Hsp90 dimerisation domains (for example the coevolving sites L641 and P684) [20]. Here, all amino acid numbers refer to the amino acid position in the reference Hsp90 sequence from *Saccharomyces cervisiae*. Group G4 included 2 amino acid sites (V260 and A287) that are close to the amphipathic loop involved in the protein client binding region [20]. Group G1 includes one amino acid in the charge linker domain (E244). Groups G8, G10, G11 and G12 include amino acids close in the sequence and in the 3D structure to the dimerization domains (T688 in G8, N673 in G10, G555 in G11 and K541 in G12) and one amino acid around L450-F492 that is surrounding the dimerization region. The two coevolving amino acids of G9 (A477 and D499) were three-dimensionally proximal to each other and to the dimerization domain as well. Application of the filter of hydrophobicity reduced the number of groups from the 13 groups identified under parsimony filtering to 7 groups, including G3, G4, G5, G7, G8, G9 and G12. Interestingly, all these groups included sites proximal to functionally important regions, except G5. Finally, groups G4, G5, G7, G8, G9, G10, G11, and G13 were detected when we applied the filter of correlation in the variances of molecular weights, with all the groups except G5 and G13 being involved or close to the dimerization domains or to the client protein binding domain.

**Table 3:** Coevolution analysis in the heat-shock protein 90.

Group of coevolution	**Amino acid sites**^a^	**Mean H(X)**^b^	**Mean H(Y)**^c^	**Mean H(X, Y)**^d^	**Mean MI**^e^
G1	E244, T458	0.4617	0.3993	0.6336	0.2275
G2	E244, K541	0.4617	0.5573	0.7610	0.2581
G3	E244, Q578	0.4617	0.3602	0.5588	0.2631
G4	V260, A287	0.3247	0.3585	0.4589	0.2243
G5	V260, I520	0.3247	0.3038	0.3605	0.2680
G6	V260, N673	0.3247	0.3220	0.4138	0.2328
G7	A287, K541	0.3585	0.5573	0.6573	0.2585
G8	L450, T688	0.5628	0.4966	0.8183	0.2411
G9	A477, D499	0.3524	0.2251	0.3524	0.2251
G10	A477, N673	0.3524	0.3220	0.4489	0.2256
G11	P482, G555	0.3452	0.4028	0.5027	0.2453
G12	F492, K541	0.3942	0.5573	0.7139	0.2376
G13	I520, G555	0.3038	0.4028	0.4768	0.2298

^a ^Amino acid sites included in each coevolution group. All sites within the same group coevolve between each other. Numbers indicate the position of the site taking as reference the sequence of *Saccharomyces cerevisae*.

^b ^Measure of mean uncertainty for the first site of the coevolving pair.

^c ^Measure of mean uncertainty for the second site of the coevolving pair.

^d ^Measure of the mean joined uncertainty for the pairs of sites within each group

^e ^Mutual Information mean value for all the possible coevolving pairs within the group.

Regarding the env protein of HIV-1, we identified 651 pairs of amino acid sites with significant MI values at the confidence level of 0.001. Application of the parsimony criterion to filter coevolving pairs, reduced the number of pairs to 10 pairs of amino acid sites organised into 3 groups of coevolution (Table 4). G1 involved 4 amino acids (L21, S640, L641 and T723) L21 is allocated in the protein gp120, while the rest of the amino acids of the remaining groups are found in the gp41 protein. Position L21 is surrounding the CD4 and gp41 binding sites, while two out of the sites found in gp41 (S640 and L641) are surrounding glycosylation site H564; we do not have relevant biological information for the position T723. Here, all amino acid numbers refer to the amino acid position in the env HXB2 reference sequence. G2 include 3 amino acids (L21, S640 and V833), with S640 and V833 being located in gp41 close to the glycosylation site H564 and T818, respectively. The last group of coevolution, G3, included 3 amino acids (T232, S640 and L641), one (T232) is located in the gp120 protein close to the glycosylation site E267. Glycosilation consists on the covering of the surface of the molecule by carbohydrates shielding therefore the antigenic exposed sites of gp120 and enabling the virus to escape the immune response of the host [21-24]. The other 2 amino acids, as explained before, belong to the gp41 protein and are surrounding gycosylation sites as well. Application of the filter of hydrophobicity reduced the number of groups from 3 to 1 group (G2), which in this case just include the pair L21 and V833. Finally, the three groups were detected when we applied the filter of correlation in the variance of the molecular weight.

**Table 4:** Coevolution analysis in the env protein of the Human Immunodeficiency Type 1 virus HIV-1.

Group of coevolution	**Amino acid sites**^a^	**Mean H(X)**^b^	**Mean H(Y)**^c^	**Mean H(X, Y)**^d^	**Mean MI**^e^
G1	L21, S640, L641, T723	0.5560	0.5527	0.8858	0.2228
G2	*L21*, S640, *V833*	0.5391	0.5510	0.8858	0.1926
G3	T232, S640, L641	0.5930	0.6066	0.8858	0.2385

^a ^Amino acid sites included in each coevolution group. All sites within the same group coevolve between each other. Numbers indicate the position of the site taking as reference the sequence of *Saccharomyces cerevisae*.

^b ^Measure of mean uncertainty for the first site of the coevolving pair.

^c ^Measure of mean uncertainty for the second site of the coevolving pair.

^d ^Measure of the mean joined uncertainty for the pairs of sites within each group

^e ^Mutual Information mean value for all the possible coevolving pairs within the group.

## Discusion

Proteins evolve (fix amino acid mutations) under rules governed by the different selective constraints imposed over their constituent amino acids. The magnitude of these constraints is a function of the relative contribution of proteins to the biological fitness of individuals in the population. Because different amino acids within a protein have different roles, they are usually under different selective pressures depending upon their importance on the protein's function or structure. This differential relationship of amino acid sites to the protein's function is translated into different levels of within-protein amino acid conservation. Amino acids that contribute equally to the protein's function or structure should theoretically correlate in their evolutionary rates. Unraveling correlated evolution between amino acids is however generally swamped in a background of historical coevolution that makes difficult disentangling both types of covariation.

Despite that most of the functionally important sites are generally restricted to change by selection, mutations studies have shown that non-conserved amino acid sites can be functionally important because of their contribution to maintain geometry-volume characteristics around important sites, their compensatory effects of mutations at important regions or their contribution to protein-protein interaction interfaces. We have previously shown that indeed most of the significant coevolutionary relationships can affect regions surrounding functionally important domains [25]. This implies that coevolving sites detected as not being proximal in the three-dimensional structure in other studies [26] may be related through their contribution to the structural/functional equilibrium of important regions in the protein. In such cases, introducing statistical filters based on biological information about the physico-chemical properties of the coevolving amino acids can significantly improve our ability to detect functionally important regions as shown in this study.

Our results based on simulated MSAs demonstrate that the accuracy of MI based methods to detect coevolution can improve by introducing statistical and biological filters *a priori *to the identification of coevolving amino acid sites. Introducing statistical filters such as those proposed previously [17] or using parsimony-informative amino acid sites as we show in this study increases SN and PPV values significantly. As we show, despite the introduction of statistical filters to the analysis of coevolution, SN and PPV still depend on the size of MSAs, mean pairwise divergence levels and strength of the coevolution signal. All these factors have intermingled effects and they should be considered prior to any coevolution analysis. The effect of the interaction of all three factors in PPV and SN is however complex and differs significantly with specific combinations of values for these factors. Fares and Travers [7] highlighted the significant effect of amino acid divergence levels and size of the MSAs on the SN of the method. They also showed that SN values of the MI based methods did not go beyond 20% when no filters were introduced. Martin and colleagues demonstrated using simulated MSAs that the contribution of background MI from finite column lengths is mitigated when MSAs comprise more than 150 sequences [17]. When analysing real protein sequences, the number of sequences in MSAs is often limited to few sequences, which make the use of MI to detect coevolution prohibitive given the large expected number of false coevolutionary relationships. We show that, the effect of the number of sequences on the variance of SN and PPV is significant but that parsimony filtering criterion employed in our coevolution analyses reduces the number of FP, increasing hence the PPV. This procedure ameliorates hence the problem of low statistical power of MI coevolutionary analyses when the number of sequences in the MSA is low. The effect of the number of sequences in MSAs becomes less important when the strength of coevolution increases. This is especially notorious when switching from MSAs comprising 50 sequences to those with 100 sequences. These results support the fact that introducing stringent filters will increase the ability of avoiding FP in the coevolutionary analyses even when the MSA sizes are limited. Furthermore, MSAs presenting high number of sequences (for example 100 sequences) present proportional increase of PPV and SN when the coevolution level increases. This means that our ability to classify sites with high MI values and their distinction from those with lower MI values is more feasible when the number of sequences is high. Consequently, these results suggest that the separation between the different types of coevolution may be more approachable in large MSA.

Parsimony criterion has been previously used successfully to identify pairs of coevolving residues involved in the interaction between two proteins [18]. Adding hydrophobicity and molecular weight as *a priori *filters for MI values we obtained significantly greater MI values compared to parsimony filtering. In addition, the size of the MSA did not have significant effects on PPV and SN. Moreover, increasing the level of coevolution did not involve a significant increase in PPV or SN when divergence levels were high. Although this approximation is similar to that performed previously [6], their approach was uniquely based on the *a posteriori *qualitative use of the biological properties of amino acids to decide whether a coevolutionary pair was important biologically. Our approach however, considers the correlation in the hydrophobicity and/or molecular weight of coevolving amino acid sites *a priori *to statistically determine their biological significance.

In summary, taking into account this study for a given protein we can theoretically interpolate the values for the size of MSAs and mean pairwise amino acid distance and know at which level of coevolution we will be able to identify real coevolving pairs. Application of our method using the different filters to identify coevolving amino acid sites within the heat-shock proteins GroEL and Hsp90 and the envelope protein env of HIV-1 clearly show the usefulness of these biological filters to identify functionally important amino acid regions. This approach was performed previously using a parametric method to detect coevolution [7] and applying it to the Hsp70-Hop-Hsp90 system [25] providing great sensitivity to detect functionally important regions. In this study we show that these filters are very useful to ameliorate the limitations of non-parametric methods to detect functional coevolution as well.

## Conclusion

The main conclusion derived from this study is that appropriate statistical as well as biological filters introduced in coevolutionary analyses can substantially reduce the false discovery rate. The number of sequences, mean pairwise divergence levels and the strength of coevolution between amino acid sites in MSAs are strong parameters affecting the ability of non-parametric methods to detect coevolution. As additional parameters affecting the accuracy of coevolution methods based on the mutual information criterion are the asymmetric phylogenies and the lack of biological filters. The high correlation in the hydrophobicities and molecular weights of closely located amino acid sites in the protein structure is testament to the importance of the biological information in these kinds of analyses. Introduction of these amino acid properties as biological filters in MI based methods appears to equalise their sensitivity to a similar range of values determined by previous simulation studies of other parametric and highly sensitive methods [7].

## Methods

### Mutual Information content

The mutual information content (MI) is a measure of our ability to predict the amino acid composition of a second amino acid site (*j*) based on the known amino acid composition of the putative coevolving site (*i*). To achieve this objective, the Shannon's entropy (H) is estimated for each of the sites considered. The entropy *H*(*X*) for a discrete random variable X (in this case the amino acid site), with each letter (amino acid) having a probability distribution (Observed frequency of an amino acid) *p*(*X*)*N *= {*p*(*x1*), *p*(*x2*),....., *p*(*xN*)} and being the sum of probabilities equal to 1, can be estimated as:

(1) $$H(X)=-\sum_{i=1}^{N}p\left(x_{i}\right)\log_{20}p\left(x_{i}\right)$$

The logarithm permits scaling H(X) so as to have H(X) = 1 when there is no uncertainty about the amino acid site composition. The mutual information is then measured taking into account the information (lack of uncertainty) for two amino acid sites (X and Y) and is calculated as:

(2) *MI*(*X*, *Y*) = *H*(*X*) + *H*(*Y*) - *H*(*X*, *Y*)

H(X, Y) is the joint entropy of two random variables (here two amino acids in the multiple sequence alignment) at amino acid sites *i *and *j *and is calculated as:

(3) $$H(X,Y)=-\sum_{i=1}^{N}\sum_{j=1}^{K}p\left(x_{i},y_{j}\right)\log_{20}p\left(x_{i},y_{j}\right)$$

Here N and K are the different elements that amino acid sites *i *and *j *could contain, respectively.

MI is a value that ranges between 0, when amino acid sites *i *and *j *evolve independently from each other, and a positive value that is proportional to the amount of evolutionary dependency between these sites.

### Filtering pairs of coevolving amino acid sites

To remove the stochastic coevolutionary signal some authors have weighted the MI value by using the amount of mutual information contained on the amino acid sites under consideration [17]. Other authors have partially accounted for the phylogenetic covariation by predefining in a qualitative way the size of groups of coevolution [13].

We have introduced two filters different from those introduced by these authors. The first filter is based on selecting pairs of coevolving amino acid sites that are parsimony informative (for example, select those amino acid sites that show at least two different amino acid each one repeated at least twice at that amino acid site in any two sequences of the MSA). Figure 1 shows an example of when an amino acid site would be considered parsimony-informative or not. Notice that the phylogenetic relationships are never accounted for in the identification of coevolving sites since the approach is purely based on the amount of patterned information present at two particular amino acid sites. Although this filter is less quantitatively sensitive than that proposed by Martin and colleagues [17] nonetheless it presents the advantage of making the detection of true positive coevolving sites less subjected to the background amino acid site variability (for example, the detection of coevolving sites does not depend on the mean level of amino acid site variability). Moreover, coevolutionary analyses are less error prone when we have asymmetric phylogenies. For example, when one branch in the tree is significantly longer than the remaining branches this branch will contain most of the evolutionary signal (this would be regarded as phylogenetic coevolution; Figure 1).

One additional filter that may improve the detection of functional coevolution between amino acid sites is that based on biological parameters. What are the relevant biological parameters is not clear and previous studies have only conducted few qualitative (non-statistical) attempts to use such filters. Among the different physico-chemical characteristics (for example, amino acid volume, geometry, molecular weight and hydrophobicity), molecular weight and hydrophobicity are the most important parameters in explaining functional links between coevolving amino acids. In general, hydrophobic amino acids will be placed in the core of the protein or in the inter-subunit interfaces in a solvent-free environment. In spite of the expected improvement of sensitivity to detect coevolution when biological information is used, methods have usually ignored this information due to the number and complex dependencies among biological properties. In theory the probability for a pair of coevolving sites to be correlated also in these two parameters is very low unless they are located in a protein region with similar hydropathy. We may however find that proximal amino acid sites in the protein structure may be correlated in their hydrophobicities or molecular weight to maintain the volume-geometry characteristics of local protein structures, affecting therefore the stability of the full protein structure. Correlations in the inter-species variation of amino acids molecular weights at neighbouring sites may uncover compensatory relationships between sequences. This kind of approximation has however the drawback of ignoring long-range distant functionally related and coevolving amino acid sites. Its conservative character would on the other hand decrease the rate of false positives.

To examine these dependencies we first tested whether amino acid sites are correlated in their hydrophobicities or molecular weight characteristics based on their three-dimensional locations in the protein structure. For such purpose, we downloaded from NCBI a dataset comprising 45,000 protein structures. We then removed redundant structures and ended with a non-redundant set of 8000 protein structures. We built a MSA for each data protein structure comprising the most similar (A maximum of 40% sequence divergence) protein sequences isolated from eukaryotic organisms. Orthologous sequences were identified using BLASTP program and taking sequences showing mutual best hits and a low probability (P < 10^-6^). We then examined the correlation in the hydrophobicities and molecular weights for each protein structure looking at all the possible pairs of amino acid sites in each structure and using the following procedure:

For each amino acid site we estimated the pairwise difference in hydrophobicity or molecular weight comparing two particular species. We called this quantity $\hat{H}Y_{ij}$. $\hat{H}Y_{ij}$ is then the estimated variation in hydrophobicity in the comparison between sequences (species) *i *and *j *at a particular amino acid site (A) of the MSA and is calculated as.

(4) $$\hat{H}Y_{ij}=\left|HY_{i}-HY_{j}\right|$$

In this work, correlation in hydrophobicity (*ρ*_Hy_) or molecular weight (*ρ*_*MW*_) between a pair of amino acid sites was measured as follows:

(5) $$\rho_{HY}=\frac{\sum_{S=1}^{T}\left[{\left(\hat{H}Y_{ij}\right)}_{AS}-\bar{H}y_{A}\right]\left[{\left(\hat{H}Y_{ij}\right)}_{BS}-\bar{H}Y_{B}\right]}{\sqrt{\sum_{S=1}^{T}{\left[{\left(\hat{H}Y_{ij}\right)}_{AS}-\bar{H}Y_{A}\right]}^{2}\sum_{S=1}^{T}{\left[{\left(\hat{H}Y_{ij}\right)}_{BS}-\bar{H}Y_{B}\right]}^{2}}}$$

Here $\hat{H}Y_{ij}$ is the estimated variation in hydrophobicity in the comparison between sequences *i *and *j *as shown in equation (4).

This calculation was repeated for all the possible pairwise comparisons in column site A. Each value $\hat{H}Y_{ij}$ is then subtracted from the mean pairwise sequence variability value for the hydrophobicity of amino acid site A. The same procedure is followed for the amino acid site B in the MSA. Both values are then multiplied and the product summed for the total number of pairwise sequence comparisons T, with T hence being:

(6) $$T=\frac{N(N-1)}{2}$$

Here, N stands for the total number of sequences in the MSA. The correlation in the variability of the molecular weight is calculated in the same way and as follows:

(7) $$\rho_{MW}=\frac{\sum_{S=1}^{T}\left[{\left(\hat{M}W_{ij}\right)}_{AS}-\bar{H}y_{A}\right]\left[{\left(\hat{M}W_{ij}\right)}_{BS}-\bar{M}W_{B}\right]}{\sqrt{\sum_{S=1}^{T}{\left[{\left(\hat{M}W_{ij}\right)}_{AS}-\bar{M}W_{A}\right]}^{2}\sum_{S=1}^{T}{\left[{\left(\hat{M}W_{ij}\right)}_{BS}-\bar{M}W_{B}\right]}^{2}}}$$

### Simulation of intra-molecular coevolution

Testing the sensitivity of methods to detect coevolution, using MSA of real proteins is tedious due to the lack of knowledge regarding intra-molecular amino acid site evolutionary and functional interactions. Forcing coevolution between known pairs of sites in simulated MSAs built on the bases of the evolutionary and physico-chemical properties of real proteins allows a better understanding of the power of methods designed for such purpose. Building MSA under realistic models of evolution is however anything but straightforward since models usually oversimplify real evolutionary processes. These limitations oblige researchers to test specific and explicit hypotheses individually and independently from others. In other words, the effect of the different factors has to be tested individually to simplify the problem discerning the main factors affecting the sensitivity of the methods.

We tested the effects of three main factors in the sensitivity to detect coevolution: i) when the strength of coevolution between two amino acid sites varies; ii) under a range of realistic mean pairwise sequence amino acid distances in MSAs; and iii) under different MSA sizes. Although the relationships between the amount of coevolution and MI values is clear, the linearity between MI and the sensitivity of the method when the coevolutionary relationships between sites changes has not been tested before. We tested this relationship using simulated MSAs presenting different coevolutionary strengths. We also examined and tested when the coevolutionary relationship between a pair of amino acids is sufficiently strong as to overcome the effect of background noise or limited MSA size on the sensitivity of the method to detect coevolution.

**Figure 7:**
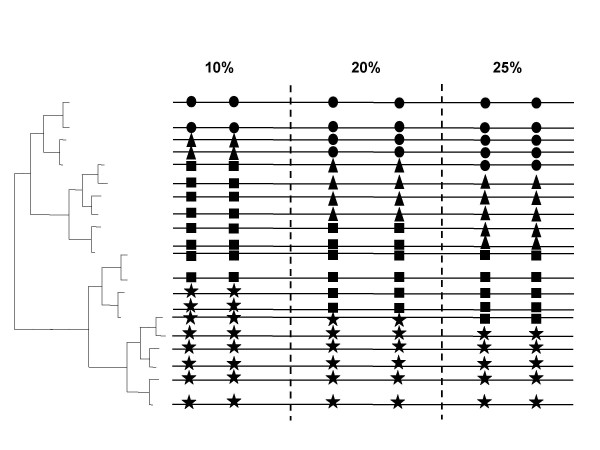
**Representation of the different types of coevolution strengths**. In this example we represent a phylogeny of 20 related sequences the amino acid states at each sequence is represented by one of the four different symbols, circles, star, sqaure and triangle. Coevolution in the simulation data set was set at three different strengths: 10% with 10 of the sequences (2 sequences) sharing the same amino acid state (circle for example), a different 10% of sequences was sharing another pattern or amino acid state (triangles), 40% another state (star) and the remaining 40% sharing the final state (square). The same rationale applied to the case of 20% as well as 25% coevolution strengths. These levels of coevolution arer considered low (yielding low Mutual Information values, the case of 10%), medium (greater MI values, 20%) and strong coevolution (the greatest possible MI values, 25%).

In order to compare the performance of our method with previous parametric and non-parametric approaches, we used the simulation procedure for divergence levels and number of sequences previously used [2,7]. The sensitivity of this method was then comparable on average to that of the non-parametric methods of Korber et al. [3], Tillier and Lui's method [13] and to that of the parametric methods of Fares and Travers [7] and Pollock et al. [2]. To test the effect that the amount of coevolution has on separating real coevolution from stochastic covariation, we forced the coevolution between five amino acid sites, and with each site presenting coevolution with all the other four. We simulated MSAs using a model similar to that devised by Pollock et al. [2] as to avoid biasing the sensitivity of our method to detect true coevolving pairs due to the method employed in the simulations (avoiding then the circularity in our approach). In one dataset we forced a pattern of 10% coevolution, which means that 10% of sequences at that site had a particular pair of amino acids, other 10% had a different pair, and then the remaining 80% contained two pairs of sites divided in 40% for one pair and 40% for the other pair (see Figure 7 for details). The other dataset contained columns showing 20% coevolution and the last dataset contained amino acid site pairs showing 25% coevolution. These dataset presented hence examples showing qualitatively weak coevolution (10%), medium coevolution (20%) and maximum (strong) coevolution (25%). Regarding mean pairwise amino acid distances, these ranged between 0.1 and 2 amino acid substitutions per site. Finally, the number of sequences in the MSA varied between 20 sequences and 100 sequences. In total we therefore simulated 3 (10%, 20% and 25% coevolution) × 3 (20, 50 and 100 sequences per MSA) × 5 (0.1, 0.2, 0.5, 1, 2 amino acid substitutions per site and tree) MSA datasets, with each one containing 100 simulated MSAs replicates. In total hence we simulated 4500 MSAs to study these three effects on the sensitivity of the method to detect coevolution. We performed all the simulations assuming a symmetric binary tree and with amino acid sites equally represented in frequency.

We used the program Evolver from the PAML package version 3.15 [27] to conduct all the primary simulations. The method of simulation used is the same as that previously published [7]. Briefly, an ancestral sequence of 200 amino acids in length was generated using the amino acid composition corresponding to the equilibrium residue frequencies in naturally occurring proteins [28]. We then evolved the ancestral sequence using a Markov chain Monte Carlo simulation along a bifurcated and symmetrical phylogenetic tree showing the divergence levels explained above. Simultaneously to this simulation we randomly chosen 10 pairs of amino acid positions and forced them to coevolve under the different coevolutionary strengths explained above.

For each one of the dataset above, we analysed the performance of the method when we filter by the parsimony-informative criterion. To show the improvement of the method to disentangle real coevolution from stochastic coevolution using biological information we also conducted the above simulations but forcing coevolving sites to correlate in their hydrophobicity or molecular weight. The number of total simulations hence was of 4500 × 3 (Figure 1). Finally, we tested the robustness of the method by measuring the effect of the different factors on the sensitivity (SN) as well as on the percentage of positive values (PPV) calculated as:

(8) $$SN=\frac{TP}{TP+FN}$$

(9) $$PPV=\frac{TP}{TP+FP}$$

Here TP, FP and FN stand for the percentage of true positives (percentage of times where we detect real pairs under coevolution), false positives and false negatives, respectively. TP refer to those pairs of amino acid sites that were forced to coevolve in the simulated data and that were detected when applying the coevolutionary analyses. We are aware of the circularity that simulations may introduce, however we have assumed that functional sites coevolve in their physico-chemical properties and that this coevolution is diluted by the stochastic noise generated throughout the evolution of proteins. In our simulations we have followed exactly this assumption introducing noise and testing the performance of the method to identify the real coevolutionary signal.

### Analysis of real data

Simulated MSAs have the disadvantage of under-representing biological information and being also "well behaved" regarding the effect of a particular factor in the analysis. We hence used a MSA for the heat-shock protein GroEL (Table including accession numbers and name of species is in Additional file 1). The ATPase molecular chaperone GroEL is found specifically in bacteria and the organelles of eukaryotic cells [29]. The multimeric protein GroEL folds 10% to 15% of slow-folding proteins, which are mostly aggregation-prone [30,31]. Each GroEL subunit is organized in three domains, including the apical, equatorial and intermediate [32,33]. Several functionally important intra-domain regions in GroEL have been previously identified (Table 1). Here we test if coevolution among sites is crucial for the functional and structural stability of GroEL. We used *groEL *gene sequences isolated from a diverse range of gamma-proteobacteria. The range of bacteria was selected in a way that allowed us testing the effect of accelerated rates of evolution on the ability of the method to detect functional coevolution. For such purpose, we selected free-living bacteria as well as endosymbiotic bacteria of the aphid insect *Buchnera aphidicola*. The effective population sizes of this endosymbiotic bacterium are subjected to strong bottlenecks during the transmission between generations. In addition, they present vertical transmission and no recombination, which ensures a high rate of slightly deleterious mutations being fixed due to genetic drift [34,35]. GroEL performs a good example to examine coevolution because the evolutionary rate is among the slowest of the endosymbiotic proteins and yet is faster than its free-living bacterium homologue. Bacteria used for this study and the accession numbers of their GroEL and GroES sequences are collected in Table 1 of supplementary information.

In the coevolutionary analyses we followed the approach recently published to detect functionally or structurally important domains [25]. In this study we focused on coevolving pairs previously detected as functionally important but also in those pairs three-dimensionally proximal to functionally important domains. The rationale behind this is that GroEL is a highly conserved molecule and most of the sites involved in ATP binding or GroES and substrates binding are very conserved as to be detected by conventional coevolutionary analyses. In these cases, pairs of amino acid sites do mainly coevolve to maintain the structural characteristics around important functional regions and consequently to maintain the conformational and functional stability of the domain [36]. Even though circular, the opposite reasoning can be used for a primary detection of functionally/structurally important protein sites or domains. We therefore used this rationale to test whether identifying coevolving pairs of sites could allow us to identify known functionally important domains due to their three-dimensional proximity. For example, when a residue was involved in inter-protein coevolution we examined whether functionally important sites nearby any of the coevolving amino acid regions (less than 8 Å distance from the functionally important region) was previously reported. If that was the case, we supported the usefulness of the method to detect functional domains. Consequently, this approach could be also valid for identifying functionally important sites that show very low evolutionary rate and that would therefore be completely ignored by standard coevolutionary analyses. We have also used additional real cases of proteins which domains are well characterised, including the env protein of the Human Immunodeficiency Virus HIV-1 group M subtypes and the Heat-shock protein 90 (Hsp90). These proteins show different divergent levels and their functional domains are well characterised and are therefore highly suitable for the purpose of this work. The sequences used for *env *gene of HIV-1 and for Hsp90 and their accession numbers are shown in Tables 2 and 3 of Supplementary Information, respectively.

Hsp90 is an ATPase molecular chaperone that assists the conformational maturation of molecules involved in signal transduction and cell cycle regulation [37-41]. The function of Hsp90 depends largely on its dimerisation and several domains can be identified in the Hsp90 linear sequence (supplemental Table 1 in [7]). The fundamental function of Hsp90 relies on the complex intra-molecular interaction between the different domains, which are poorly understood. The *env *gene of HIV-1 is the gene yielding the functionally important proteins gp120 and gp41 that are involved in host cell recognition, binding and entry [42-44]. The coordinated function of gp120 and gp41 is translated into a complex evolutionary patterns observed in the *env *gene (i.e. [45,46]) and even more complex coevolutionary patterns [25,47]. Here we apply the improved method to detect coevolutionary patterns in the *env *gene of the HIV-1 subtype.

## Authors' contributions

MAF has conceived the study, designed the computational and statistical analyses, conducted the statistical analyses, written the corresponding software and written the manuscript. FMC has run the coevolutionary analyses in the simulated data and in the real proteins and implemented part of the computational code and SO has conducted some of the analyses and partially funded the study.

## Supplementary Material

Additional file 1 Accession numbers for the protein sequences used in the analysis of co-evolution in the real data set. Click here for file
