# Supplementary material for: Reducing the false positive rate in the non-parametric analysis of molecular coevolution
Source: BMC Evol Biol. 2008 Apr 10;8:106. doi: 10.1186/1471-2148-8-106 (PMC2362121; doi:10.1186/1471-2148-8-106)
Supplement: Additional file 1 — Accession numbers for the protein sequences used in the analysis of co-evolution in the real data set. [file 1471-2148-8-106-S1.doc]

**Supplementary Information**

Table1. Accession numbers for the sequences of the protein GroEL. Sequences from free-living bacteria and primary symbionts (PS) or secondary symbionts (SS) of insects are included

| Specie | Accession Number |
| --- | --- |
| *Escherichia coli* | X07850 |
| *Salmonella typhimurium* | U01039 |
| *Klebsiella pneumoniae* | U81143 |
| *Enterobacter aerogenes* | AB008141 |
| *Erwinia caratovora* | AB008152 |
| *Tuberolachnus salignus* | AJ439086 |
| *Thelaxes suberi PS* | AJ439085 |
| *Rhopalosiphum padi PS* | U77380 |
| *Schizaphis graminum PS* | AF008210 |
| *Acyrthosiphon pisum PS* | X61150 |
| *Myzus persicae PS* | AF003957 |
| *Pterocomma populeum PS* | AJ439083 |
| *Tetraneura caerulescens PS* | AJ439084 |
| *Sodalis glossinidius SS* | AF404511 |
| *Wigglesworthia glossinidia PS* | AF321516 |
| *Hemophilus influenzae* | NP_438701 |
| *Amoeba proteus PS* | M86549 |

Table 2. Accession numbers for the heat-shock protein 90 (Hsp90).

| **Organism** | **Protein Acc Number** |
| --- | --- |
| Homo sapiens | NP_001017963 |
| Pan troglodytes | XP_001160832 |
| Macaca fascicularis (Macaque) | BAE01934 |
| Sus scrofa (pig) | NP_999138 |
| Bos taurus | NP_001012688 |
| Rattus norvegicus | NP_786937 |
| Mus musculus | NP_034610 |
| Equus caballus (horse) | BAB20777 |
| Gallus gallus | CAA30251 |
| Xenopus tropicalis | NP_001016282 |
| Xenopus laevis | AAH72998 |
| Danio rerio | NP_001038538 |
| Dicentrarchus labrax | AAQ95586 |
| Astyanax mexicanus (Mexican tetra) | AAO52675 |
| Oncorhynchus tshawytscha (Chinook salmon) | AAB49983 |
| Spodoptera frugiperda | AAG44630 |
| Mamestra brassicae | BAF03554 |
| Opistophthalmus carinatus (African scorpion) | AAQ94359 |
| Bombyx mori | NP_001036876 |
| Chilo suppressalis | BAE44307 |
| Chiromantes haematocheir | AAS19788 |
| Antheraea yamamai | BAD15163 |
| Apis mellifera | XP_623939 |
| Plutella xylostella | BAE48742 |
| Locusta migratoria | AAS45246 |
| Haliotis tuberculata | CAK95235 |
| Tribolium castaneum | XP_967904 |
| Bemisia tabaci | AAZ17403 |
| Aedes aegypti | EAT36186 |
| Drosophila melanogaster | NP_523899 |
| Delia antiqua | CAI64494 |
| Drosophila auraria | AAB58358 |
| Chlamys farreri | AAR11781 |
| Tetraodon nigroviridis | CAG03540 |
| Liriomyza sativae | AAW49253 |
| Ceratitis capitata | CAJ28987 |
| Anopheles albimanus | AAB05638 |
| Liriomyza huidobrensis | AAW49252 |
| Brugia pahangi | CAA06694 |
| Dendronephthya klunzingeri | CAC38753 |
| Heterodera glycines | AAO14563 |
| Caenorhabditis elegans | CAA27441 |
| Schistosoma japonicum | NP_999808 |
| Oryza sativa (japonica cultivar-group) | BAD04054 |
| Arabidopsis thaliana | NP_200411 |
| Triticum aestivum | ABG57075 |
| Solanum lycopersicum | AAB01376 |
| Hordeum vulgare | AAP87284 |
| Hevea brasiliensis | AAQ08597 |
| Ipomoea nil | AAA33748 |
| Nicotiana benthamiana | AAR12194 |
| Solanum tuberosum | ABB55365 |
| Nicotiana tabacum | BAE97400 |
| Zea mays | AAB26482 |
| Schizosaccharomyces pombe | NP_594365 |
| Candida albicans | XP_721353 |
| Saccharomyces cerevisiae | NP_013911 |
| Plasmodium falciparum 3D7 | NP_704028 |
| Entamoeba histolytica HM-1:IMSS | EAL47778 |
| Dictyostelium discoideum AX4 | XP_647482 |
| Theileria parva strain Muguga | XP_764810 |
| Cryptosporidium hominis | EAL35500 |

Table 3. Accession numbers for the sequences of the envelope protein-coding gene of HIV-1.

| Subtype | Representative sequences (GenBank accession no.) |
| --- | --- |
|  | |
| A1 | [AF004885](http://jvi.asm.org/cgi/external_ref?access_num=AF004885&link_type=GEN), [AF457080](http://jvi.asm.org/cgi/external_ref?access_num=AF457080&link_type=GEN) , [AF069673](http://jvi.asm.org/cgi/external_ref?access_num=AF069673&link_type=GEN), [AF19327](http://jvi.asm.org/cgi/external_ref?access_num=AF19327&link_type=GEN), [AB098333](http://jvi.asm.org/cgi/external_ref?access_num=AB098333&link_type=GEN), [AF457075](http://jvi.asm.org/cgi/external_ref?access_num=AF457075&link_type=GEN), [AF484478](http://jvi.asm.org/cgi/external_ref?access_num=AF484478&link_type=GEN), [U51190](http://jvi.asm.org/cgi/external_ref?access_num=U51190&link_type=GEN) |
| A2 | [AF286237](http://jvi.asm.org/cgi/external_ref?access_num=AF286237&link_type=GEN), [AF286238](http://jvi.asm.org/cgi/external_ref?access_num=AF286238&link_type=GEN) |
| B | [U63632](http://jvi.asm.org/cgi/external_ref?access_num=U63632&link_type=GEN), [AY037270](http://jvi.asm.org/cgi/external_ref?access_num=AY037270&link_type=GEN) , [U69589](http://jvi.asm.org/cgi/external_ref?access_num=U69589&link_type=GEN), [AF042102](http://jvi.asm.org/cgi/external_ref?access_num=AF042102&link_type=GEN) |
| C | [AF110967](http://jvi.asm.org/cgi/external_ref?access_num=AF110967&link_type=GEN), [AF110971](http://jvi.asm.org/cgi/external_ref?access_num=AF110971&link_type=GEN) , [AF443091](http://jvi.asm.org/cgi/external_ref?access_num=AF443091&link_type=GEN), [AF286227](http://jvi.asm.org/cgi/external_ref?access_num=AF286227&link_type=GEN) |
| D | [U88822](http://jvi.asm.org/cgi/external_ref?access_num=U88822&link_type=GEN), [AY237166](http://jvi.asm.org/cgi/external_ref?access_num=AY237166&link_type=GEN) , [AF484505](http://jvi.asm.org/cgi/external_ref?access_num=AF484505&link_type=GEN), [AF484519](http://jvi.asm.org/cgi/external_ref?access_num=AF484519&link_type=GEN) |
| F1 | [AF077336](http://jvi.asm.org/cgi/external_ref?access_num=AF077336&link_type=GEN), [AF005494](http://jvi.asm.org/cgi/external_ref?access_num=AF005494&link_type=GEN) , [AJ249238](http://jvi.asm.org/cgi/external_ref?access_num=AJ249238&link_type=GEN), [AY173957](http://jvi.asm.org/cgi/external_ref?access_num=AY173957&link_type=GEN) |
| F2 | [AJ249236](http://jvi.asm.org/cgi/external_ref?access_num=AJ249236&link_type=GEN), [AF377956](http://jvi.asm.org/cgi/external_ref?access_num=AF377956&link_type=GEN) , [AJ249237](http://jvi.asm.org/cgi/external_ref?access_num=AJ249237&link_type=GEN) |
| G | [AF061642](http://jvi.asm.org/cgi/external_ref?access_num=AF061642&link_type=GEN), [AF061640](http://jvi.asm.org/cgi/external_ref?access_num=AF061640&link_type=GEN) , [AF084936](http://jvi.asm.org/cgi/external_ref?access_num=AF084936&link_type=GEN), [AF423760](http://jvi.asm.org/cgi/external_ref?access_num=AF423760&link_type=GEN) |
| H | [AF190128](http://jvi.asm.org/cgi/external_ref?access_num=AF190128&link_type=GEN), [AF005496](http://jvi.asm.org/cgi/external_ref?access_num=AF005496&link_type=GEN) , [AF190127](http://jvi.asm.org/cgi/external_ref?access_num=AF190127&link_type=GEN) |
| J | [AF082394](http://jvi.asm.org/cgi/external_ref?access_num=AF082394&link_type=GEN), [AF082395](http://jvi.asm.org/cgi/external_ref?access_num=AF082395&link_type=GEN) |
| K | [AJ249235](http://jvi.asm.org/cgi/external_ref?access_num=AJ249235&link_type=GEN), [AJ249239](http://jvi.asm.org/cgi/external_ref?access_num=AJ249239&link_type=GEN) |
